# Biosensor-Integrated Virtual Reality for Cognitive Behavioral Therapy in Psychosis: A Systematic Review of a New Therapeutic Frontier

**DOI:** 10.3390/bios16050265

**Published:** 2026-05-03

**Authors:** Aristomenis G. Alevizopoulos, Georgios G. Anastasiou, Iakovos Kritikos, Maria Alevizopoulou, Georgios A. Alevizopoulos

**Affiliations:** 1 Department of Psychiatry, Agioi Anargyroi General Oncological Hospital of Kifisia, National and Kapodistrian University of Athens, 14564 Athens, Greece; galev@uoa.gr; 2 BIOSIM Laboratory, School of Electrical and Computer Engineering, National Technical University of Athens, 15780 Athens, Greece; el19112@mail.ntua.gr; 3 Global Business School for Health, University College London, London WC1E 6BT, UK; iakovos.kritikos.24@ucl.ac.uk; 4 School of Medicine, European University Cyprus, 2404 Nicosia, Cyprus; ma222294@students.euc.ac.cy

**Keywords:** virtual reality (VR), biosensors, psychosis, delusions, hallucinations, cognitive behavioral therapy, digital therapeutics, personalized medicine, avatar therapy, VR-assisted technologies

## Abstract

Psychosis presents significant treatment challenges, and standard Cognitive Behavioral Therapy for psychosis often faces limitations due to patient engagement issues and reliance on subjective self-reporting. The integration of Virtual Reality (VR), physiological biosensors, and artificial intelligence offers a transformative opportunity to address these challenges. A systematic review and meta-analysis were conducted in accordance with PRISMA guidelines. A thorough literature search was performed across seven databases. Twelve randomized controlled trials involving 1504 participants were included to assess VR-assisted CBT, VR treatment, and AVATAR therapy. Meta-analyses showed that VR interventions significantly decreased auditory verbal hallucinations (pooled SMD = −0.24, *p* = 0.0011) and paranoid thoughts (SMD = −0.26, *p* < 0.0001) compared to control conditions. This review supports integrating multi-modal biosensors to collect real-time, objective physiological data. Such integration enables the development of AI-driven, closed-loop systems that dynamically adjust the virtual environment based on the patient’s physiological state. VR-assisted therapies effectively reduce positive symptoms of psychosis. Incorporating biosensors is a crucial step toward a data-driven approach for personalized, closed-loop psychiatric care. Future efforts should focus on large-scale clinical trials, biomarker validation, and robust ethical frameworks to ensure safe and effective implementation.

## 1. Introduction

Psychosis is a serious mental illness characterized by a disconnection from reality. People experiencing psychosis may have delusions, which are false beliefs that are not based in reality, and hallucinations, which involve seeing, hearing, or feeling things that aren’t there. These experiences can be distressing and cause significant impairment in daily functioning. Psychosis can occur as a symptom of several mental disorders, either chronic, such as schizophrenia, bipolar disorder, or major depression, or acute. It can be brought on by substance use, sleep deprivation, or certain medical conditions [1]. The delusional perceptions experienced by psychotic patients often have a significant impact on their social and occupational functioning, ultimately reducing their overall quality of life [2]. These delusions can disrupt and even destroy the interpersonal relationships that individuals have built with others. A classic example is the patient’s persistent belief that others are threatening them [3]. The social impact of psychosis is exacerbated when the onset occurs at a younger age, as this doesn’t allow the patient to cope with the core psychotic symptoms that could potentially lessen the impact of the disorder [4].

### 1.1. The Challenge of Functional Recovery in Psychosis

Despite the availability of evidence-based pharmacological and psychotherapeutic treatments, achieving sustained functional recovery in individuals with psychosis remains a formidable challenge even nowadays. Research has shown that almost 30% of schizophrenia patients, the most common chronic psychotic disease, are resistant to treatment with at least two different antipsychotics [5,6]. The complex nature of the disorder, characterized by positive symptoms such as delusions and hallucinations, negative symptoms like avolition and anhedonia [7], and significant cognitive impairment, makes it difficult to diagnose because of the broad spectrum and the different combinations of symptoms a patient can present with. In essence, patients suffering from psychosis have lost the ability to create functional thought patterns and have replaced them with negative thoughts and maladaptive behaviors. Many interventions demonstrate variable effectiveness, and a subset of patients shows resistance to gaining insight into their condition, further complicating the therapeutic process [8,9]. This clinical reality underscores the pressing need to explore and apply novel technologies to enhance existing treatment options and to offer new pathways to recovery [8].

### 1.2. Cognitive Behavioral Therapy for Psychosis as the Standard of Care and Its Limitations

Cognitive Behavioral Therapy (CBT) is a psychotherapeutic approach that focuses on identifying and modifying these dysfunctional thought patterns (cognitions) and behaviors. It is based on the premise that our thoughts, feelings, and behaviors are interrelated and that by changing our approach, our emotional well-being and functioning can be dramatically improved. CBT is typically structured and goal-oriented, using specific techniques to challenge and reframe negative thoughts, develop coping strategies, and change behavior patterns. CBT is often delivered in a time-limited format, focusing on teaching clients’ skills they can use independently to manage their symptoms and improve their overall quality of life. Individuals learn to identify and challenge distorted beliefs, develop coping strategies, and prevent relapse by learning to recognize early warning signs of psychosis [10,11].

Cognitive Behavioral Therapy for psychosis (CBTp) is recognized as the “gold standard” non-pharmacological intervention, demonstrating efficacy in alleviating key symptoms and substantially improving social functioning [10,12]. CBTp operates on the premise that by identifying and modifying dysfunctional thought patterns and maladaptive behaviors, individuals can improve their emotional well-being and functioning [12]. However, the real-world application of CBTp is hampered by several significant limitations. Patients often struggle with exposure-based social training, and the therapy’s reliance on self-report is compromised by the very cognitive biases it seeks to treat. Furthermore, limited implementation in routine traditional clinical settings, low patient engagement, and the stigma associated with seeking mental healthcare act as substantial barriers to accessing this effective treatment [11,13].

### 1.3. The Emergence of Digital Therapeutics

Various studies over the years have shown that patients in mental healthcare have substantial difficulty accepting who they are and in differentiating their view of themselves from how others perceive them. Self-discrepancy theory states that any discomfort a person feels is related to the difference between their actual self and their ideal self or their perceived self in situations involving others. Disturbances in self-awareness, such as false beliefs, thoughts, or stimulus interpretations, can strongly affect a person’s emotions and integrated responses. This point is relevant to relapse prevention, as changing these emotional responses can help prevent a person from slipping back into previous habits. If a method can be developed to assist at this cognitive level, it would be a major stride in a technologically innovative approach to help people with mental illnesses [14,15,16].

The rise of digital mental health interventions represents a promising approach to enhance both the quality of and access to care [17]. Among these technologies, Virtual Reality (VR) stands out as a uniquely powerful clinical tool, offering new opportunities for assessment, intervention, and research that are difficult or impossible to achieve with traditional methods [18]. VR is a relatively old technology, invented by Morton Heilig in 1957, although the term was coined by Jaron Lanier 30 years later. VR technology describes a computer-generated 3D environment that allows the user to create immersive, ecologically valid environments where patients can learn and practice skills in scenarios that are more realistic than traditional clinic-based exercises [19]. These technologies have wide applications in healthcare, including surgeries, diagnostics, treatment, rehabilitation, training, and education [20]. The sense of reality induced by VR has been shown to elicit physical and psychological responses similar to real-life experiences, but unlike traditional assessments conducted in controlled laboratory or clinical settings, especially in the case of phobias [21]. In addition, VR technology can be integrated and combined with other promising digital therapies, like artificial intelligence (AI) and AI-driven speech, to enhance cognitive screening for many neuropsychiatric diseases, primarily Alzheimer’s disease [22].

An experimental study tested the first VR social environment used for a drug-dependent population, and it is considered to be a successful study as real-time data can be gathered in a realistic simulation without risks to patients in social situations of the real world. This could be further developed with modern technology to monitor biometric changes in real conditions of the real world. With the intent to maximize cost-efficiency, this method would have the potential to replace traditional face-to-face methods of monitoring, only if there are no significant differences in the monitored data [23].

This technological push is occurring in parallel with a significant clinical pull. The demand for more effective and accessible psychosis treatments, driven by factors such as mental health professionals’ shortages and the need for scalable solutions, is converging with the market-driven supply of affordable [24,25], high-fidelity consumer VR headsets and wearable biosensors [8]. These dynamic shifts the primary challenge from one of technological possibility to one of clinical validation, rigorous implementation science, and careful ethical oversight. The question is no longer if these tools can be used in mental healthcare, but how they can be deployed safely and effectively.

### 1.4. The Use of Biosensors in Medicine

The development of modern medicine is linked to the capacity to measure and quantify the body’s very complicated physiological processes. Biosensors, which convert a biological response into a measurable signal, are the pinnacles of that capability. They provide real-time data for processes of cardiac, neurologic, and metabolic activity, changing the diagnostics from a qualitative observation and a report-dependent technique to a quantitative-based technique. The physiological systems they monitor are not segregated; they are very intertwined and are drastically impacted by an individual’s psychological state and thus, they need to work simultaneously. Consequently, the data generated by these sensors are now being rerouted as powerful, objective biomarkers for mental health. The main types of biosensors that are readily available are electrocardiography (EKG), electroencephalography (EEG), electromyography (EMG), sweat sensors, and electrodermal activity sensors [26]. These devices have already-established medical applications in specialties like cardiology and neurology, but now their use is gaining increasing significance in psychiatry. These technologies are connected by their ability to translate body language into strict, numerical data. Thus, many more biosensors have been created that only work in conjunction with other biosensors, like pupil dilation detection sensors [27]. Biosensors are essential for the development of sophisticated, bio-integrated therapeutic devices. Additionally, VR environments, through special headsets, can be combined with many biosensors, to assist in the diagnosis and treatment of many diseases, including psychiatric ones. Researchers have utilized adaptive VR environments that dynamically change in response to real-time feedback from biosensors to diagnose anxiety disorders and to treat phobias, such as arachnophobia and a fear of heights [21,28,29]. On the other hand, the cost of developing and implementing these types of systems should not be underestimated. Given the level of expertise and equipment required, it would be difficult to make such multifunctional systems available, at this time, at a cost that would be affordable to many patients and providers. However, a dramatic reduction in the cost of equipment is expected soon, because of all the tools developed to facilitate medical care during the COVID-19 pandemic [30]. This review has a dual aim. First, to systematically evaluate the current efficacy, usage, and tolerability of VR-assisted CBT in treating positive symptoms of psychosis and schizophrenia spectrum disorders. Second, to narratively synthesize how multi-modal biosensors can be integrated into these validated VR protocols to create next-generation, closed-loop digital therapeutics.

## 2. Materials and Methods

We conducted a comprehensive systematic review in accordance with the Preferred Reporting Items for Systematic Reviews and Meta-Analyses (PRISMA) statement [31]. The review process involved collecting, analyzing, and summarizing information from published primary clinical data. Therefore, no ethical approval or individual participant consent was required. For transparency, the review protocol and data are registered and available in the PROSPERO database under registration number CRD420251181566.

Because fully integrated VR-biosensor systems for psychosis are currently in the theoretical and prototyping phases, the systematic review portion of this paper focuses strictly on establishing the baseline efficacy of VR interventions. Concurrently, the biosensor integration is presented as a forward-looking narrative synthesis, bridging established technological capabilities from related fields (e.g., anxiety disorders) with the specific clinical gaps identified in psychosis treatment. This hybrid approach was chosen to ensure the transparency, comprehensiveness, and reproducibility of the literature identification process while allowing narrative integration of findings from diverse study designs.

### 2.1. Search Strategy

This systematic review analyses the current literature in VR-assisted technologies to diagnose, manipulate, and normalize perceptual distortions in psychosis. It also compares their efficacy with that of standard treatments. A comprehensive literature search was performed using the PubMed/Medline, Web of Science, PsycINFO, Embase, Google Scholar, Scopus, and CINAHL databases, starting in March 2026. Search terms (keywords) included “virtual reality”, “bio-sensors”, “computer simulation”, “computer-assisted therapy”, “psychosis”, “delusions”, “hallucinations”, “thought disturbance”, and “CBT treatment- therapy intervention” on their own, as well as using different combinations of keywords with Boolean operators. While this broad search strategy, which included individual keywords without always specifying ‘VR’ or ‘AR’, initially generated a large volume of non-specific results, it was deliberately chosen to ensure a comprehensive capture of the literature and maximize literature recall. By prioritizing sensitivity, we aimed to minimize selection bias and ensure the comprehensive identification of all relevant clinical trials about our research question. The search also included systematic reviews on the topic found by checking the reference lists of the papers first analyzed (but were not included in the selected studies), as well as papers from the references of the articles that appeared in our initial search with the above keywords and their combinations. There were no publication dates or country exclusions. All papers reviewed were in English.

### 2.2. Data Extraction and Screening

All studies identified from the selected five databases were collected independently by two reviewers (A.A. and G.G.A) in the citation management tool “Zotero”. After removing all duplicates using the DOI finder, the same two reviewers independently screened the titles, abstracts, and full-text availability. Afterwards, the reviewers screened the full text of the eligible studies in accordance with our strict inclusion and exclusion criteria, to finalize the selection.

Reviewers performed data extraction using standardized Excel spreadsheets and the Zotero management tool. The extracted data from the included studies comprised general study characteristics, baseline participant demographics, and the pre-specified efficacy and safety outcomes. The general characteristics of the trials included the first author, publication year, type of intervention, control group chosen, sample size, target area, study duration and follow-up, primary findings, and effect sizes. The baseline characteristics of patients included age, gender, ethnicity, sample size, primary psychiatric diagnosis, and the country in which the study took place.

### 2.3. Inclusion and Exclusion Criteria

The inclusion criteria were original peer-reviewed articles from academic journals involving (a) individuals meeting the DSM-4, DSM-5 or ICD-10 diagnostic criteria for psychosis or schizophrenia spectrum disorders [32]; (b) use of VR-assisted cognitive behavioral therapy (VR-CBT), VR-assisted treatment (VRT), or AVATAR therapy for psychosis; (c) randomized controlled trial (RCT) study design; (d) capture of quantitative data; (e) main symptoms being treated were positive ones; (f) clinical and/or general population participants. No age limitations were applied to the participants. We excluded articles that were not in English or articles for which only the abstract was available online. Additionally, observational studies were excluded because of their inherent risk for bias.

### 2.4. Results of the Literature Search

**Figure 1:**
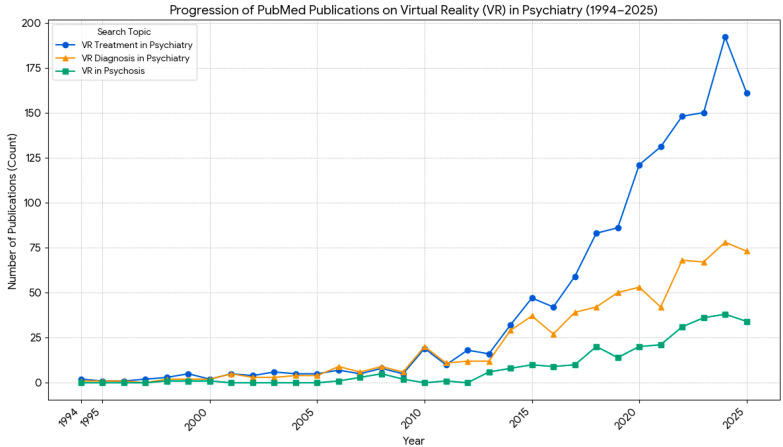
Trends in research articles indexed in the PubMed/Medline database from 1994 to 2025 on VR in the field of psychiatry. Blue line (circles): VR-assisted treatment; Orange line (triangles): VR-assisted diagnosis; Green line (squares): VR use in psychosis and schizophrenia.

**Figure 2:**
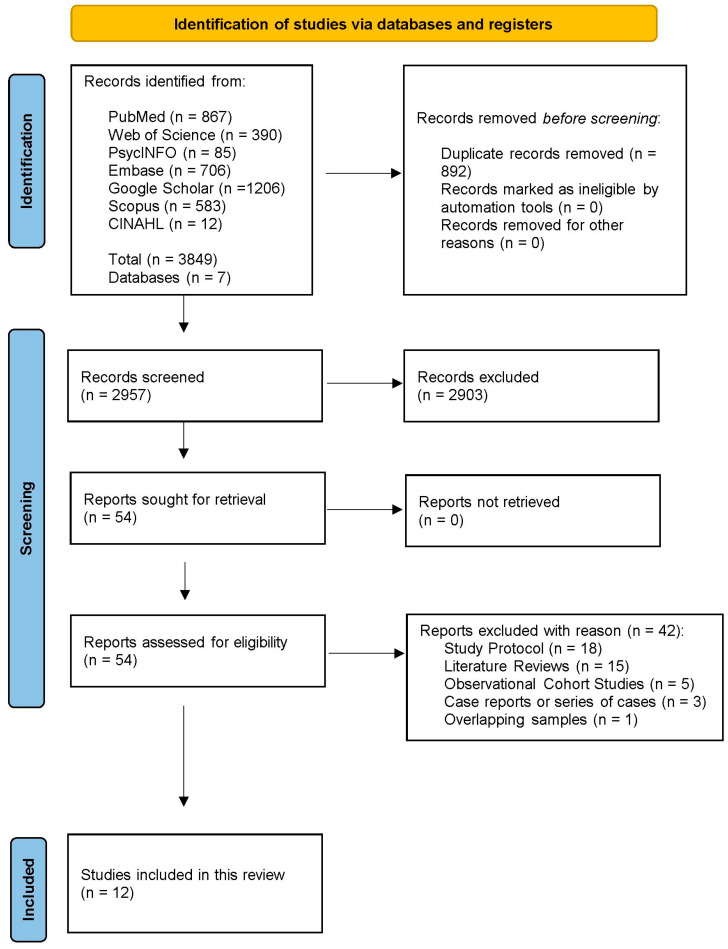
PRISMA 2020 flow diagram for study inclusion.

The database search yielded a total of 3849 records from 7 different databases (867 PubMed/Medline, 390 Web of Science, 85 PsycINFO, 1206 Google Scholar, 583 Scopus, and 12 CINAHL). The records removed before screening totaled 892, because they were duplicates, leaving 2957 records to be screened based on title and abstract. During the initial title and abstract screening phase, 2903 records were excluded because they did not meet our strict clinical relevance criteria. Although Figure 1 demonstrates hundreds of articles broadly relating to VR use in psychosis and schizophrenia, the vast majority of these were excluded from our final analysis because they were not RCTs, did not target positive symptoms (such as auditory hallucinations or delusions), did not capture quantitative data, or focused purely on observational/diagnostic outcomes rather than active therapeutic interventions. From the remaining 54 full-text reports, 42 were excluded for being study protocols (*n* = 18), literature reviews (*n* = 15), observational cohort studies (*n* = 5), case reports or series of cases (*n* = 3), and studies with overlapping samples (*n* = 1). Finally, 12 clinical studies were included in the review that studied outcomes of VR treatments in humans. The selection process is illustrated according to the PRISMA flow chart in Figure 2.

## 3. Results

There is a growing amount of literature on the use of VR, and increasingly, VR integrated with biosensors, to manipulate and normalize perceptual distortions in psychosis. As shown in Figure 1, there has been a significant increase over the last decade in both VR-assisted diagnosis and treatment of psychiatric disorders. Still, the research, especially in psychosis and schizophrenia spectrum disorders, didn’t increase proportionally. By region, North America dominates augmented reality (AR) and virtual reality (VR) in the healthcare market, accounting for the largest revenue share of 49.64% in 2022. Other countries with significant research activity in this area include China, Japan, India, Australia, and New Zealand [33].

### 3.1. Characteristics of Included Studies

This review summarizes and synthesizes findings from 12 randomized controlled trials (RCTs), investigating the use of VR-assisted cognitive behavioral therapy, VR-assisted treatment (excluding VR-CBT), or AVATAR therapy for the treatment of positive symptoms of psychosis and schizophrenia spectrum disorders [34,35,36,37,38,39,40,41,42,43,44,45].

Collectively, the studies involved a total of 1.504 participants. Of these, 497 received AVATAR therapy, 192 received VR-assisted treatment (VRT), 373 received VR-CBT, 39 received VR mental relaxation therapy (VRMR), 245 received standard CBT, 53 underwent VR exposure as a control, and 356 received only treatment as usual (TAU), which included antipsychotic medications, supportive counseling, and being waitlisted for VR-CBT.

The key findings and information of these studies are presented in Table 1, while the population demographics and relevant data are presented in Table 2.

### 3.2. Risk of Bias and Quality Assessment

**Figure 3:**
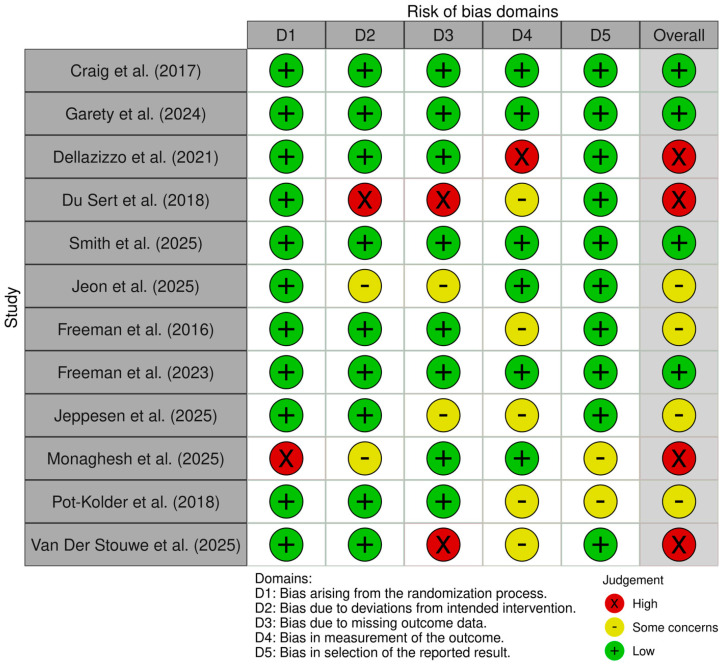
Risk of bias summary for the 12 included randomized controlled trials evaluating VR-CBT, VR Therapy, and AVATAR Therapy, utilizing the RoB-2 tool [34,35,36,37,38,39,40,41,42,43,44,45].

**Figure 4:**
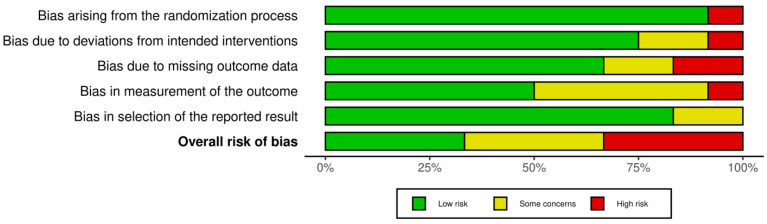
Risk of bias graph displaying the weighted percentages of low risk, some concerns, and high risk across all five domains for the evaluated clinical trials.

To ensure transparency and to accurately assess the quality of the included RCTs, the review team used the Revised Cochrane risk-of-bias tool for randomized trials (RoB 2) [46]. The tool comprises and assesses 5 domains regarding the risk of bias: bias arising from the randomization process (D1); bias due to deviations from the intended intervention (D2); bias due to missing outcome data (D3); bias in the measurement of the outcome (D4); and bias in the selection of the reported result (D5). The judgment of each domain was classified as either low risk, some concerns, or high risk of bias. The results of our quality assessment are illustrated in Figure 3 and Figure 4 and were created using the online “RobVis” tool (webapp version) [47].

Four studies [34,35,38,41] had low concerns, four studies [39,40,42,44] had some concerns and four studies [36,37,43,45] had high concerns regarding the overall risk of introducing bias. Du Sert et al. (2018) and Van Der Stouwe et al. (2025) had high concerns regarding missing outcome data, not provided by the research teams [37,45]. Monaghesh et al. (2025) had great concerns regarding the randomization process, since the trial was based in a single center [43].

### 3.3. Auditory Verbal Hallucinations (AVH) and AVATAR Therapy (AT)

The application of VR for treating hallucinations is a promising area of research. The predominant VR-based approach is AVATAR therapy, in which a patient co-designs a digital avatar within a 2D environment that represents their primary persecutory voice, and then engages in a therapeutic dialogue with it, supported by a therapist [35,48,49]. Thus, the patient can regain control of the situation. To create the appropriate space, a voice-morphing program and virtual reality environment are employed, allowing therapists to transform their voice to sound just like the AVH the patient hears [50,51].

In the two RCTs focusing on AVATAR therapy, either brief or extended, vs. TAU, 305 participants experienced AT for the treatment of AVHs [34,35]. These trials represent a logical and critical progression in the clinical evaluation of a novel psychological intervention. The first study, Craig et al. [34], often referred to as AVATAR1, was a single-site, single-blind RCT designed to establish the initial efficacy of AVATAR therapy, while Garety et al. [35], or AVATAR2, was a larger, more pragmatic phase 2/3 multi-site RCT, designed to evaluate the effectiveness of the treatment in a more diverse, real-world scenario with more therapists and centers participating.

While both AT and VRT described in these papers are founded on the same basic therapeutic principle, which is a dialogue-based, relational therapy whereby the patient communicates with a digital representation of their primary distressing voice, the basic difference lies in the technology employed to present it. The early AVATAR 1 and 2 therapy trials employed a plain 2D computer screen to present the avatar to the patient. On the other hand, VRT interventions represent a technical evolution of this method through the use of immersive 3D VR delivered via a head-mounted display (VR headset). This move to a more immersive medium is based on the working hypothesis that a higher sense of presence in the virtual environment could create a more intense emotional and behavioral reaction, thereby possibly boosting the therapeutic effect of the dialogue. Building on this foundation, the integration of biosensors is a critical next step to enable data-driven personalization. These sensors will deliver objective, real-time physiological data and seamlessly interact with the VR environment. Rather than simply heightening the realism of the simulation, this closed-loop system can automatically adjust the therapeutic challenge based on the patient’s actual stress levels, ensuring optimal exposure, improved safety, and a highly tailored therapeutic response.

All the identified studies targeted AVH as opposed to non-verbal auditory hallucinations [36,37,38,39,40,41]. This is because the voice software and the therapist cannot mimic the abstract nature of the non-verbal hallucinations patients experience, like music or bangs [52]. Also, all AT and VRT studies used the auditory hallucinations subscale (AHS) of the PSYRATS (Psychotic Symptom Rating Scales), a specialized clinical tool that provides a detailed, multidimensional measure of the severity of specific psychotic symptoms, to assess the outcome of their interventions [53,54,55]. PSYRATS-AH consists of 11 items, each rated on a 5-point scale (0–4), with a minimum score of 0 and a maximum score of 44, to measure severity, allowing for the even comparison of the five trials.

Craig et al. [34] based their primary efficacy outcomes on an “intention to treat” basis using linear mixed-effects models. At the 12-week follow-up mark, the reduction in the PSYRATS-AH total score (0–44) was significantly greater for AVATAR therapy compared to TAU (supportive counselling). The adjusted mean difference was −3.82 (SE = 1.47), with a 95% CI of −6.70 to −0.94 and *p* = 0.0093. In addition, AT had a Cohen’s d (Cd) of approximately 0.8 while TAU typically achieves a modest effect of Cd = 0.2. Interestingly, on the second assessment of the two groups at the 24-week mark, the difference was no longer statistically significant, with an adjusted mean difference of −1.55 (SE = 1.80) and a 95% CI of −5.09 to 1.98 and *p* = 0.39.

The AVATAR 2 trial [35] had 3 groups in a 1:1:1 ratio, splitting the intervention group of AT from the last trial into two with different “dosage” groups: an AVATAR-Brief (AV-BRF) that underwent 6 sessions, and an AVATAR-Extended (AV-EXT) that underwent 12 sessions of AT. Both intervention groups received additional treatment as usual, while the third control-nonintervention group received only TAU. This allowed the researcher to additionally compare the two AT groups to control and to each other to find the optimal number of sessions needed for AT to have the most cost-effective results. The mean number of sessions attended was 5.11 (SD 2.42) for AV-BRF and 8.18 (SD 4.43) for AV-EXT. The primary analysis was again conducted using mixed-effects models on the “intention to treat” sample, with a significance level adjusted to *p* ≤ 0.035$ to account for multiple comparisons. At 16 weeks, both AV-BRF and AV-EXT were significantly better than TAU at the PSYRATS-AH total score (0–44). At TAU vs. AV-BRF, the difference in therapeutic effect was −2.04 (SE = 0.853) with a 95% CI of −3.836 to −0.239, *p* = 0.017, and Cd = 0.45. At TAU vs. AV-EXT, the difference in therapeutic effect was −2.32 (SE = 0.894) with a 95% CI of −4.208 to −0.438, *p* = 0.009, and Cd = 0.52. At 28 weeks, the effect of the AT groups was not maintained. At TAU vs. AV-BRF, the difference in therapeutic effect was −1.61 (SE = 1.256) with a 95% CI of −4.260 to −1.036, *p* = 0.199, and Cd = 0.36. At TAU vs. AV-EXT, the difference in therapeutic effect was −1.87 (SE = 1.138) with a 95% CI of −4.274 to −0.526, *p* = 0.100, and Cd = 0.42. But the extended version of the therapy showed a more durable effect on the key secondary outcome of voice frequency.

The results indicate a statistical drop in efficacy from the previous efficacy trial to this larger effectiveness trial. The effect on the primary outcome in AVATAR1 was large (Cd = 0.8), while in AVATAR2, the effect sizes were small to moderate (Cd = 0.38 for AV-BRF and Cd = 0.58 for AV-EXT at 16 weeks). This reduction in effect size is to be expected in the transition from a highly controlled efficacy trial to a more pragmatic effectiveness trial and could be due to several factors, including the use of a less strict control condition and greater variability of delivery across multiple sites and therapists. Furthermore, the results indicate a visible contradiction between efficacy and practicability. The longer, personalized AV-EXT treatment protocol exerted a more significant impact over a broader spectrum, particularly on voice frequency and delusions. However, its completion rate (58%) was much lower than that of AV-BRF (82%). Adding more intense, trauma-focused elements to AV-EXT would serve both to enhance its therapeutic effectiveness and to make it harder for other participants to finish, so appropriate patient selection or stepped-care in clinical practice is recommended.

The second trial examined several potential baseline moderators of treatment effect. The prespecified hypothesis, although the complexity of voice characterization was expected to moderate the outcome, was not supported. The analysis found very few significant moderation effects overall. Those that met the significance threshold included an interaction for AV-EXT between treatment effect and Index of Multiple Deprivation (IMD) quintiles, and an interaction for AV-BRF with age at first hearing voices, where an earlier onset was associated with larger treatment effects.

### 3.4. VRT for AVHs

A VRT protocol is an evolution of the classic 2D AT, which is a relational therapy for troubling voices in psychosis. VRT focuses on therapist-led conversations with a digital avatar that speaks. In contrast to AT, VRT employs immersive 3D VR technology [56]. A phase-II randomized partial cross-over trial (*n* = 19) in treatment-resistant schizophrenia compared 7 weeks of VRT with treatment-as-usual (TAU; antipsychotics plus routine clinical contact). VRT produced significant improvements in AVH severity, depressive symptoms, and quality of life, with effects maintained at 3-month follow-up. Reductions were especially marked for distress associated with persecutory voices, with a very large effect size (d = 1.2) [37].

Dellazizzo et al. (2021) [36] compared VRT vs. CBT for psychosis (CBTp) (*n* = 74) in treatment-resistant schizophrenia, evaluating short- and long-term efficacy over a 1-year follow-up. Both VRT and CBTp led to significant reductions in AVH severity and depressive symptoms [36]. VRT was not statistically superior on AVH outcomes, but showed larger effect sizes for overall AVH (d =1.080 vs. 0.555 for CBT) and suggested superiority on affective symptoms, with additional benefits for persecutory beliefs and quality of life. Effects were maintained up to 12 months [36].

In the Challenge trial (*n* = 270), Smith et al. (2025) compared immersive Challenge-VRT vs. enhanced TAU in adults with schizophrenia and treatment-resistant AVH, defined as ≥3 months with poor response to antipsychotics [38]. At 12 weeks, Challenge-VRT significantly reduced overall AVH severity on PSYRATS-AH compared with enhanced TAU (adjusted mean difference −2.26, Cohen’s d 0.27) and significantly reduced AVH frequency at 12 and 24 weeks [38]. The choice to recruit treatment-resistant patients may be due to the method being rather new, with relatively sparse knowledge on the risk of adverse events.

**Figure 5:**
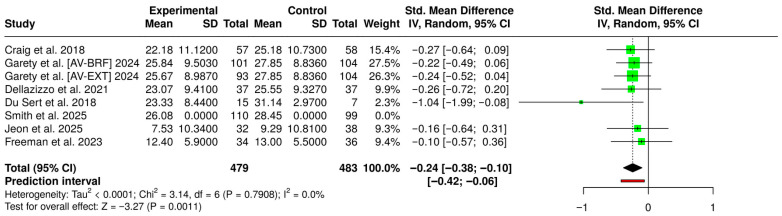
Forest Plot of SMD for the post-treatment PSYRATS-AH scores, comparing experimental interventions (AT and VRT) to control conditions [34,35,36,37,38,39,41].

Because all identified AT and VRT studies used the same measurement tool, the PSYRATS-AH scale, it is easier to evaluate the interventions. The PSYRATS-AH standardizes symptom severity into a total score, creating a consistent metric that allows for even and reliable comparison of results across the five trials [53]. The results reported by the seven studies for AVH, post treatment, are illustrated in Figure 5. Using a random-effects model, by MetaAnalysisOnline.com, with the inverse-variance method to compare the standardized mean difference (SMD), the analysis found a statistically significant difference between the two cohorts; the pooled SMD was −0.24 (95% CI: [−0.38, −0.10], *p* = 0.0011). The calculation pools data from a substantial number of participants, comparing 479 individuals in the experimental groups against 483 in the control groups. The included trials demonstrate exceptional consistency, as indicated by an I^2^ value of 0.0% and a *p*-value of 0.7908 for heterogeneity, indicating that the effect sizes across cohorts were uniform in magnitude and direction [57]. Because a lower PSYRATS-AH score indicates less severe symptoms, this negative SMD mathematically favors the experimental group, proving the treatment’s efficacy over the control.

### 3.5. Delusions and Paranoia

VR-assisted CBT has been evaluated against waiting-list controls and against standard CBTp for paranoia and related persecutory ideas and delusions, often using the Green (Revised) Paranoid Thoughts Scale (GPTS/R-GPTS) as a key outcome [58,59]. Results were mixed but generally favorable effects versus control, with modest advantages over standard CBT on paranoid ideation.

Mediation analyses, performed by Pot-Kolder et al. (2018), indicated that reductions in safety behaviours and social cognition problems explained substantial portions of the change in paranoid ideation (34% and 19%, respectively) [44]. These outcomes reflect decreases in persecutory ideas and delusions of reference, both in-the-moment (ESM measures) and on more global scales of ideas of persecution and social reference.

In the RCT by Van Der Stouwe et al. (2025) (*n* = 98), VR-CBTp was compared head-to-head with standard CBTp in people with psychotic-spectrum disorders and paranoid ideation [45]. Both groups showed substantial reductions in momentary paranoia (ES ≈ 0.65), with greater reduction for VR-CBTp at post treatment (interaction ES ≈ 0.62). Secondary paranoia measures confirmed improvement (ES 0.66–1.15), but between-group differences on these secondary scales were not robust. VR-CBTp showed advantages in some related domains. There were greater reductions in safety behaviour and depression and greater self-esteem gains post treatment; self-esteem and anxiety effects persisted at follow-up. Although this trial did not use the GPTS as the primary endpoint, it indicates that VR-CBTp may yield somewhat stronger acute reductions in real-life paranoid ideation and related processes than standard CBTp.

Jeppesen et al. (2025) compared 10 sessions of VR-CBTp vs. CBTp on top of usual care, using the GPTS/R-GPTS score, and found no significant between-group difference at endpoint (effect estimate 2% in favor of VR-CBTp; d = 0.04; *p* = 0.77) [42]. Both treatments reduced persecutory ideas; however, scores in both groups were low (≤8.7) across follow-ups, below the recommended cutoff of ≥11 for persecutory delusions, suggesting a floor effect limiting scope for further improvement on the GPTS. Thus, when paranoia is already in the mild range, VR-CBTp does not outperform CBTp.

Monaghesh et al. (2025) [43] also compared VR-CBT with traditional CBT. In this RCT, both groups improved, but VR-CBT showed significantly greater reductions in GPTS paranoia (*p* < 0.001) [43]. Percentage GPTS reduction: ~14.1% for VR-CBT vs. 6.6% for CBT. VR-CBT also produced larger reductions in PANSS positive symptoms (capturing delusions and hallucinations) and greater gains in Theory of Mind (Eyes Test). Here, VR-CBT more effectively reduced paranoid thoughts (GPTS) and general positive psychotic symptoms than standard CBT.

A parallel-group RCT (*n* = 78) by Jeon et al. (2025) specifically targeted persecutory ideas/delusions of reference in unstable psychosis outpatients using VR therapy [39]. Patients were randomized to VR-treatment (voice-containing social scenes) or VR-control (muted versions or neutral nature scenes) and assessed pre/post with R-GPTS as primary outcomes. Path analysis indicated that fear of negative evaluation and depressive symptoms mediated treatment effects. The authors conclude that VR therapy effectively reduces delusions in young, stable psychosis patients with mild, tolerable side effects and call for the development of more varied content for older populations.

**Figure 6:**
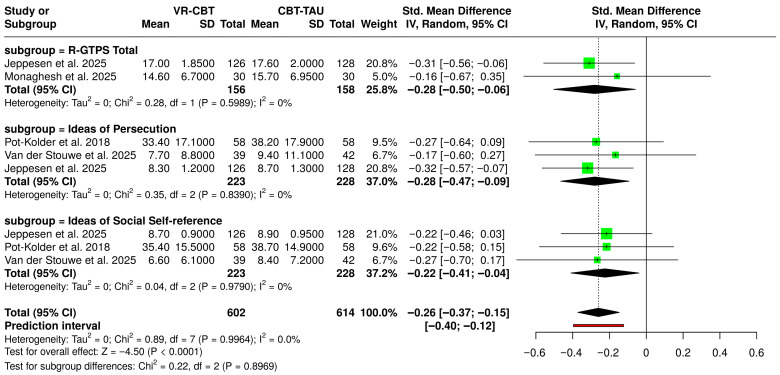
Forest Plot of SMD for the post-treatment GPTS/R-GPTS scores, comparing the efficacy of VR-CBT versus standard CBT and Treatment as Usual [42,43,44,45].

All studies focusing on paranoia reported the GPTS/R-GPTS scores of their participants post treatment. Thus, allowing for an even comparison between the RCTs, as illustrated in Figure 6. To evaluate the efficacy of VR-CBT compared to standard CBT-TAU on symptoms of paranoia and social self-reference, a pooled meta-analysis was conducted [57]. As illustrated in the forest plot, the analysis of 602 experimental participants and 614 control participants demonstrated a highly statistically significant overall effect favoring the VR-CBT intervention (SMD = −0.26, 95% CI [−0.37, −0.15], *p* < 0.0001). Subgroup analyses revealed consistent and robust therapeutic advantages for VR-CBT across all measured domains, specifically the R-GTPS Total (SMD = −0.28, 95% CI [−0.50, −0.06]), Ideas of Persecution (SMD = −0.28, 95% CI [−0.47, −0.09]), and Ideas of Social Self-reference (SMD = −0.22, 95% CI [−0.41, −0.04]). Notably, the included trials exhibited uniform consistency, with no observable statistical heterogeneity across the overall studies (I^2^ = 0.0%, *p* = 0.9964) and no significant variance between the specific subgroups (Chi^2^ = 0.22, *p* = 0.8969). Collectively, these findings provide strong empirical evidence that VR-CBT is statistically superior to standard CBT-TAU in reducing targeted paranoid ideations.

### 3.6. Safety and Tolerability

Treatment was generally well tolerated, though six serious adverse events potentially related to the intervention occurred (five hospitalizations for exacerbated AVH, one self-harm episode) in a population with long-standing severe symptoms. Completion was high (79%), and delivery by local clinicians with remote supervision supports real-world feasibility and scalability. Overall, the trial concludes that immersive VRT has short-term efficacy in reducing AVH severity in treatment-resistant schizophrenia, reinforcing evidence for avatar-based VR approaches [38].

Adverse effects were mostly described as VR sickness, with high frequency but mild severity. The authors conclude that VR therapy effectively reduces delusions in young, stable psychosis patients with mild, tolerable side effects and call for the development of more varied content for older populations [39]. Cybersickness was measured using the Simulation Sickness Questionnaire, which was administered to the VR-CBT group during each session. Jeppesen et al. (2025) used an unweighted approach to calculate total scores [42]. Mean total score at session 1 was 6.75 (95% CI 5.38–8.12) and mean total score at session 2 was 6.10 (95% CI 4.80–7.40) [42,60].

The frequent reporting of VR sickness, coupled with the potential for exacerbated distress and serious adverse events (such as hospitalizations for exacerbated AVH), highlights a critical gap in these current VR protocols: the reliance on subjective post-session reporting rather than real-time objective monitoring. The safety data from these early trials strongly underscores the necessity of integrating physiological biosensors. By continuously tracking a patient’s autonomic arousal and stress markers during VR exposure, clinicians could predict and prevent therapeutic flooding and adverse events before they escalate.

## 4. Discussion

### 4.1. The Value of VR-Assisted Therapies

While previous meta-analyses have established the general efficacy of VR interventions in reducing delusions, paranoia, and AVH, this systematic review provides a novel contribution by synthesizing this robust baseline evidence with a comprehensive framework for the integration of biosensors. We uniquely highlight how current VR therapies can evolve from subjective, exposure-based tools into objective, data-driven digital therapeutics [49,61,62,63]. Moreover, the VR intervention led to superior improvements in secondary outcomes, including the reduction in safety behaviors and depressive symptoms, suggesting that the immersive environment may accelerate or enhance specific therapeutic mechanisms central to CBTp [45]. However, there seems to be a discrepancy in the efficacy between delusions and hallucinations, suggesting that the therapeutic mechanisms engaged by current VR platforms are more aligned with belief modification than with altering perceptual disturbances [61].

Crucially, VR interventions appear to target the underlying cognitive mechanisms of social dysfunction. For example, four sessions of VR-CBT were significantly superior to traditional CBT in improving Theory of Mind, which is the ability to infer others’ mental states, as measured by the Reading the Mind in the Eyes Test. This improvement in a core social-cognitive process was directly associated with a greater reduction in paranoia, providing a clear link between enhancing social cognition and ameliorating positive symptoms [43,64].

### 4.2. The Engagement Paradox and Patient Adherence

VR is often promoted for its immersive and engaging qualities, with the assumption that it will improve patient motivation and treatment adherence [18]. However, high-quality evidence from a real-world clinical trial presents a critical challenge to this narrative. The 2025 multicenter RCT comparing VR-CBTp to standard CBTp reported a substantially higher treatment dropout rate in the VR group (37.0%) compared to the standard CBTp group (24.0%) [45]. This “engagement paradox” forces a more nuanced understanding. While therapeutically potent, the highly immersive and potentially stressful nature of VR-based exposure may be more demanding or aversive for a significant subset of patients with psychosis, leading to higher attrition. This finding has profound clinical implications, suggesting that patient selection, psychoeducation, and careful management of in-session distress are critical factors for the successful implementation of VR-CBTp. It refutes the simplistic notion that more immersion is always better and highlights a crucial area where objective monitoring could play a role.

### 4.3. Integrating Biosensors with VR Therapeutics

The limitations of subjective self-report and the challenges of managing patient distress during immersive therapy necessitate a move toward objective, data-driven interventions. Integrating physiological biosensors with VR systems is not merely an incremental enhancement but a crucial evolution required to make these therapies safer, more precise, and objectively measurable.

Psychiatry has long been constrained by its reliance on subjective patient accounts and clinician observations, a limitation that emerging technologies are poised to address. In psychosis, this challenge is amplified by the presence of cognitive biases and impaired insight, which can distort self-reported experiences. Asking a patient to self-assess their anxiety level during an immersive VR session can be both disruptive to the therapeutic experience and yield unreliable data [65,66].

Biosensors offer a direct window into a patient’s physiological state, providing objective, continuous, and real-time measures of their response to therapy. This aligns with a broader movement in psychiatry toward using objective biomarkers, from neuroimaging to physiological signals, to enable earlier detection, more precise diagnosis, and personalized treatment strategies [26,67]. When combined with VR, this approach becomes particularly powerful. The VR environment can serve as a standardized “stress test” or emotional probe, presenting precisely controlled and replicable scenarios while biosensors capture the resulting physiological and behavioral responses [8]. This allows clinicians to move beyond a patient’s qualitative description of distress to a quantitative measurement of their psychophysiological reaction to specific triggers.

It is crucial to emphasize that while the individual components, such as VR headsets, physiological biosensors, and AI algorithms, do currently exist, their fully integrated, real-time adaptive application for psychosis treatment, as described here, remains largely hypothetical and represents a visionary future research direction rather than an established clinical reality. Furthermore, achieving this integration faces significant practical implementation challenges. Continuous data streaming requires robust data security protocols, high-bandwidth infrastructure for real-time AI processing without latency, and rigorous clinical validation to ensure the dynamic adjustments are both safe and therapeutically effective. However, overcoming these hurdles to utilize continuous data streams is justified because it moves psychiatric assessment beyond episodic, subjective reporting into a realm where physiological biomarkers can objectively predict symptom exacerbation or treatment response in real time.

#### 4.3.1. A Multi-Modal Biosensing Toolkit for Immersive Psychiatry

A wide array of non-invasive sensors can be integrated with VR systems to create a rich, multi-modal picture of a patient’s state. Recent technological advancements, such as the use of nanomaterials to enhance sensor sensitivity and new coatings to improve the longevity of implantable devices, continue to expand these capabilities [8,67]. The most relevant technologies for psychosis are summarized in Table 3.

#### 4.3.2. Closed-Loop Systems: Towards Real-Time, Data-Driven Therapeutic Adaptation

The ultimate goal of integrating biosensors is to create “closed-loop” therapeutic systems. In such a system, the multi-modal data stream from the sensors is fed in real time to a processing engine, which uses machine learning (ML) or artificial intelligence (AI) algorithms to interpret the signals and infer the patient’s internal state (e.g., anxiety, cognitive overload) [66]. This inference then automatically and dynamically adapts the VR environment to optimize the therapeutic challenge. For example, if a patient’s combined EDA and HRV data indicate an overwhelming anxiety response to a virtual crowd, the system could automatically reduce the number of avatars or alter their behavior to be less confrontational, preventing the patient from becoming flooded and disengaging from the therapy.

AI and ML are critical to this vision, as they can learn to recognize complex patterns across multiple data streams and classify emotional states with high accuracy, far surpassing what is possible with single-sensor analysis [66]. This capability allows for the automation of core therapeutic processes like graded exposure, ensuring the experience remains within the patient’s “window of tolerance” while being guided and overseen by a clinician. Over time, these continuous data streams could contribute to a personalized “digital phenotype” of a patient’s illness, providing a far richer and more dynamic understanding of their symptom patterns and triggers than is possible with traditional, episodic clinical assessments.

#### 4.3.3. Implementation Challenges and Clinical Justification

Continuous physiological data streams offer utility far beyond theoretical closed-loop systems by shifting VR therapy from a reactive to a predictive model. In psychosis, where cognitive biases often compromise subjective reporting, objective data, such as sudden drops in heart rate variability, can identify subconscious triggers before a patient verbally reports distress. Mapping these responses allows clinicians to define individualized “psychophysiological biosignatures,” guiding highly targeted cognitive restructuring that standard CBTp cannot achieve.

However, transitioning these integrated systems to routine practice presents substantial implementation challenges. Technically, immersive VR involves physical movement that introduces significant noise into sensitive recordings (e.g., EEG, EKG), requiring robust real-time artifact-rejection algorithms and expensive, low-latency computational infrastructure. Clinically, continuous monitoring in a population prone to persecutory delusions demands an extraordinarily delicate approach. The act of attaching wearable sensors or discussing data tracking could inadvertently exacerbate paranoia or become integrated into delusional frameworks. Consequently, co-designing unobtrusive hardware with patient advisory groups and establishing strict data privacy protocols are mandatory steps.

While VR environments, biosensors, and machine learning algorithms exist independently, their seamless fusion into an adaptive clinical tool remains largely hypothetical. How to broadly implement and validate such integrated systems is a critical future research frontier, rather than an immediate clinical reality.

Ultimately, the core question of how to broadly implement and clinically validate such integrated systems remains largely unanswered by the current evidence base. While the individual components, VR environments, wearable biosensors, and machine learning algorithms are readily available, their seamless fusion into a validated, real-time adaptive clinical tool for psychosis remains a highly promising, yet fundamentally hypothetical, future research frontier rather than an immediate clinical reality.

## 5. Future Directions

The convergence of VR, biosensors, and CBTp marks a pivotal moment in the evolution of psychiatric care. This technology offers the potential to transform the treatment of psychosis from a practice reliant on subjective report and generalized protocols to a data-driven science of personalized, adaptive intervention. To realize this potential, the field must move forward with a clear conceptual model and a rigorous, collaborative research agenda.

### 5.1. A Synthesized Model of Biosensor-Integrated VR-CBTp

A conceptual model for a fully integrated therapeutic ecosystem can guide future development. This system would consist of several interconnected components:

1. The Patient: The individual at the center of the experience, immersed in the VR environment.
2. The VR Simulation: A library of evidence-based, targeted therapeutic scenarios that can be tailored to the patient’s specific delusional themes or social anxieties.
3. The Multi-Modal Biosensor Array: A suite of integrated, non-invasive sensors (e.g., EEG, HRV, eye-tracking) capturing a continuous stream of physiological and behavioral data.
4. The AI/ML Analysis Engine: A sophisticated computational core that processes the high-dimensional data in real-time to infer the patient’s internal state (e.g., level of arousal, cognitive load, valence of emotional response, attentional focus).
5. The Real-Time Feedback Loop: An adaptive mechanism that uses the output from the analysis engine to dynamically modify the VR simulation to maintain an optimal level of therapeutic challenge, titrating exposure based on objective data.
6. The Clinician Dashboard: An intuitive interface that provides the therapist with a synthesized, longitudinal view of the patient’s objective and subjective data, highlighting patterns, tracking progress, and informing clinical decision-making both within and between sessions.

### 5.2. A Research Roadmap for the Next Decade

To bridge the gap from this conceptual model to clinical reality, a concerted and strategic research effort is required. The following areas should be prioritized:

Definitive Clinical Trials: There is an urgent need for large-scale, methodologically rigorous, multicenter RCTs to establish the efficacy and long-term effectiveness of these integrated systems. Critically, these trials should employ a three-arm design, comparing integrated VR-biosensor systems against both VR-CBTp without biosensors and standard treatment-as-usual. This design is essential to isolate and quantify the specific added value of the real-time objective data.

Biomarker Development and Validation: Research must move beyond crude measures of “stress” or “anxiety” and focus on identifying and validating specific psychophysiological “biosignatures” of core psychotic processes within VR. For example, studies could aim to identify the unique patterns of eye-tracking and autonomic arousal that signify the triggering of a paranoid belief, or the EEG correlates of successful cognitive reappraisal. Validating these biomarkers is essential for creating truly mechanism-based interventions.

Implementation Science and Health Economics: Parallel to efficacy trials, implementation science research is crucial. This includes co-designing systems with patients and clinicians to maximize usability, acceptability, and engagement, thereby addressing the “engagement paradox”. Furthermore, rigorous health-economic analyses are needed to establish the cost-effectiveness of these technologies, which will be critical for securing reimbursement and driving adoption within healthcare systems.

Proactive Ethical and Regulatory Standards: The field must proactively develop clear ethical guidelines and regulatory standards for the clinical use of these powerful tools in vulnerable populations. This requires a multi-stakeholder collaboration involving researchers, clinicians, ethicists, regulatory bodies, technology companies, and, most importantly, individuals with lived experience of psychosis.

## 6. Strengths and Limitations

### 6.1. Strengths

Our study has several strengths. First, our review is comprehensive and includes a large sample size of 1504 participants across 12 RCTs. Additionally, we adhered strictly to the PRISMA guidelines and ensured transparency by pre-registering the review protocol in the PROSPERO database. To maintain high methodological rigor, we exclusively included RCTs and excluded observational studies due to their inherent risk of bias. We also utilized the validated RoB 2-tool to accurately assess the quality of the included studies. Furthermore, our quantitative analysis benefited from the use of standardized clinical tools across the included trials, such as the PSYRATS-AH and GPTS/R-GPTS scales, which allowed for consistent evaluation and resulted in exceptional statistical consistency with no heterogeneity among the pooled cohorts. Finally, to our knowledge, this is one of the first reviews to thoroughly explore the emerging frontier of integrating multi-modal biosensors with VR-CBTp to facilitate objective, real-time clinical assessments.

### 6.2. Limitations

Our study also has some limitations that should be acknowledged. First, our search criteria excluded articles that were not in English and those not published in peer-reviewed academic journals, which may introduce language and publication bias. Second, the section of this review focusing on the use of biosensors to support VR training had to be presented narratively rather than quantitatively, due to a significant lack of original RCT research on this novel topic. Third, the included studies showed variability in their risk of bias. Specifically, while four studies had a low risk, eight studies raised “some” or “high” concerns, largely due to missing outcome data and potential flaws in the randomization processes of single-center trials. Furthermore, there were substantial variations in the technological formats of the interventions (ranging from 2D computer screens to immersive 3D VR headsets) and in treatment dosages (brief versus extended sessions), which complicate direct comparisons of efficacy. Finally, the heterogeneity in participant baseline characteristics, spanning from young, stable psychosis outpatients to individuals with long-standing, treatment-resistant schizophrenia, means that the findings should be interpreted with caution regarding their broader clinical generalizability.

## 7. Conclusions

VR-assisted interventions effectively reduce positive symptoms in psychosis by offering safe, immersive environments for social and cognitive practice. However, the risk of immersion-induced stress, which can lead to treatment dropout, underscores the need for careful patient selection and in-session distress management.

Integrating multi-modal biosensors addresses the limitations of subjective self-reporting by providing continuous, objective data on a patient’s physiological and emotional states. This integration paves the way for AI-driven, “closed-loop” systems that can dynamically adjust the VR environment to maintain an optimal therapeutic challenge without overwhelming the patient.

While the convergence of VR and biosensors holds the potential to revolutionize psychosis care into a highly personalized and precise model, future large-scale, rigorous RCTs are essential. Moving forward, the field must focus on establishing long-term effectiveness, validating specific psychophysiological biomarkers, and addressing the ethical and data privacy challenges inherent in these novel technologies.

## Figures and Tables

**Table 1:** Summary of the included studies. Cd = Cohen’s d; PSYRATS-AH = Psychotic Symptoms Rating Scales-Auditory Hallucinations subscale; VR = virtual reality; CBT = cognitive behavioral therapy; TAU = treatment as usual; VR-CBT = virtual reality cognitive behavioral therapy; CBTp = cognitive behavioral therapy for psychosis; VRMR = virtual reality mental relaxation; VRT = virtual reality therapy.

Author (Year)	Intervention	Control Group	Population	Primary Findings	Effect Size
Craig et al. (2017) [34]	AVATAR Therapy	TAU (supportive counseling)	150	AVATAR therapy was significantly more effective in reducing AVH severity at 16 weeks but not at 24 weeks.	Cd = 0.8
Garety et al. (2024) [35]	AVATAR Therapy	TAU (N/A)	345	Both AV-BRF and AV-EXT were superior to TAU at 16 weeks, but not at 28 weeks.	At 16 weeks: AV-BRF(Cd) = 0.38, AV-EXT(Cd) = 0.58 At 28 weeks: AV-BRF(Cd) = 0.22, AV-EXT(Cd) = 0.38
Dellazizzo et al. (2021) [36]	VRT	CBT	74	Both interventions were effective. VRT was not statistically superior to CBT, but achieved a numerically larger effect.	VRT: Cd = 1.08 CBT: Cd = 0.555
Du Sert et al. (2018) [37]	VRT	TAU (antipsychotics)	15	VRT produced significant improvements in AVH severity, particularly distress, which were maintained at 3-month follow-up.	PSYRATS-AH Total: Cd = 1.0 PSYRATS-AH Distress: Cd = 1.2
Smith et al. (2025) [38]	VRT	TAU (supportive counseling)	215	Challenge-VRT significantly reduced AVH severity compared to TAU.	At 12 weeks: Adjusted Mean Difference = −2.26, Cd = 0.27
Jeon et al. (2025) [39]	VRT	VR-control	70	VRT showed a significant improvement in PSYRATS-D scores compared to VR-control.	Cd = 0.865, for positive symptoms
Freeman et al. (2016) [40]	VR-CBT	VR-control	30	VR-CBT led to significantly greater reductions in delusional conviction.	Cd = 1.3
Freeman et al. (2023) [41]	VR-CBT	VRMR	77	No significant difference between the two active VR interventions. Both groups showed large improvements.	VR-CBT(Cd) = 1.6 VRMR(Cd) = 1.3
Jeppesen et al. (2025) [42]	VR-CBT	CBT	254	VR-CBTp was not superior to standard CBTp in reducing paranoia.	Cd = 0.04 (non-significant)
Monaghesh et al. (2025) [43]	VR-CBT	CBT	60	VR-CBT was significantly superior to traditional CBT.	Cd = 0.25
Pot-Kolder et al. (2018) [44]	VR-CBT	TAU (waitlist)	116	Significant reduction in Momentary Paranoia vs. control. No immediate effect on Time with Others.	Paranoia (Cd) = 1.49
Van Der Stouwe et al. (2025) [45]	VR-CBT	CBT	98	Both groups improved. VR-CBTp showed a significantly greater reduction in Momentary Paranoia than standard CBTp.	Effect size between VR-CBT and CBT was 0.62 favoring VR-CBT

**Table 2:** Baseline participant demographics and relevant clinical data across the included studies. ^a^ = Institute of Psychiatry, Psychology & Neuroscience (King’s College London); University College London; University of Manchester; University of Glasgow, ^b^ = VIRTU Research Group; Aalborg University Hospital; Psychiatric Outpatient Services Esbjerg, ^c^ = Jeonbuk National University Hospital; Kyungpook National University Hospital; Chonnam National University Hospital; Catholic University of Korea Seoul St. Mary’s Hospital; Yeungnam University Medical Center; Inje University Haeundae Paik Hospital, ^d^ = Oxford Health NHS Foundation Trust; Berkshire Healthcare NHS Foundation Trust; Northamptonshire Healthcare NHS Foundation Trust; Milton Keynes NHS Foundation Trust, ^e^ = Altrecht; GGZ Delfland; GGZ Noord-Holland-Noord; Katholieke Universiteit Leuven; Parnassia; ProPersona; Rivierduinen; University Medical Center Groningen; SD = standard deviation.

Author (Year)	Country Setting	Size (Male)	Age in Years (SD)	Ethnicity	Main Diagnosis	Intervention (Male)	Control (Male)
Craig et al. (2017) [34]	United Kingdom, South London and Maudsley NHS trust	150 (102)	42.7 (±10.7)	White = 58, Black = 54, Asian = 5, Other = 33	Paranoid schizophrenia = 115	75 (57)	75 (45)
Garety et al. (2024) [35]	United Kingdom, university trial sites^a^	345 (212)	39.61 (±13.26)	White = 203, Black = 57, South Asian = 27	Schizophrenia = 151	230 (143)	115 (69)
Dellazizzo et al. (2021) [36]	Canada, Institut Universitaire en Santé Mentale de Montréal	74 (56)	42.5 (±12.7)	Caucasian = 61, Visible minorities = 13	Schizophrenia = 57	37 (29)	37 (27)
Du Sert et al. (2018) [37]	Canada, Institut Universitaire en Santé Mentale de Montréal	15 (10)	42.9 (±12.4)	Caucasian = 13, Visible minorities = 2	Schizophrenia = 12	15 (10), partial cross-over	15 (10), partial cross-over
Smith et al. (2025) [38]	Denmark, out-patient psychiatric clinics^b^	270 (105)	32.83 (±11.9)	N/A	Schizophrenia = 249	140 (53)	130 (52)
Jeon et al. (2025) [39]	South Korea, out-patient psychiatric clinics^c^	70 (39)	30.01 (±8.12)	N/A	Schizophrenia = 52	32 (17)	38 (22)
Freeman et al. (2016) [40]	United Kingdom, Oxford Health NHS trust	30 (16)	- VR-CBT: 42.1 (±13.4) - VR-control: 40.6 (±14.4)	White = 29, Mixed = 1	Schizophrenia = 10	15 (10)	15 (6)
Freeman et al. (2023) [41]	United Kingdom, National Health Service trusts^d^	80 (49)	40.3 (±13.1)	White = 64, Black = 6, South Asian = 4, Chinese = 1, Other = 5	Schizophrenia = 36	VR-CBT: 39 (25)	VRMR: 41 (24)
Jeppesen et al. (2025) [42]	Denmark, capital region and north Denmark region	254 (146)	26.8 (22.8–33.1)	N/A	Schizophrenia = 184	126 (54)	128 (54)
Monaghesh et al. (2025) [43]	Iran, Razi Hospital, Tabriz	60 (36)	- VR-CBT: 30.8 - CBT: 32.1	N/A	Schizophrenia = 60	30 (19)	30 (17)
Pot-Kolder et al. (2018) [44]	Netherlands, mental health centers	116 (82)	- VR-CBT: 36.5 - TAU: 39.5	Dutch = 40, Other = 76	Schizophrenia = 95	58 (40)	58 (42)
Van Der Stouwe et al. (2025) [45]	Netherlands and Belgium, mental health centers^e^	98 (72)	- VR-CBT: 35.5 (±12.9) - CBT: 36.1 (±12.3)	Dutch = 79, Other = 19	Unspecified schizophrenia spectrum and other psychotic disorder = 41	48 (33)	50 (39)

**Table 3:** Multi-modal Biosensors for VR-integrated Psychiatry. VR = virtual reality; EKG = electrocardiography; HRV = heart rate variability; EEG = electroencephalography; EDA = electrodermal activity.

Sensor Type	Physiological Target	Clinical Application in VR Psychosis Treatment
EKG/HRV	Cardiac Activity	Monitoring baseline arousal and heart-rate variability responses to auditory verbal hallucinations during AVATAR therapy.
EEG	Neurologic Activity	Assessing cognitive load and neural correlates of belief modification during exposure.
EDA/Sweat Sensors	Electrodermal Activity	Quantifying acute stress and sympathetic nervous system arousal in real-time to prevent therapeutic flooding.
Pupillometry/Eye-tracking	Pupil Dilation & Gaze	Measuring attentional focus, avoidance behaviors, and subconscious responses to persecutory virtual stimuli.

## Data Availability

Not applicable.

## References

[1] Lieberman J.A., First M.B. (2018). Psychotic Disorders. N. Engl. J. Med..

[2] Bobes J., Alegría A.A., Saiz-Gonzalez M.D., Barber I., Pérez J.L., Saiz-Ruiz J. (2010). Change in Psychiatrists’ Attitudes towards the Physical Health Care of Patients with Schizophrenia Coinciding with the Dissemination of the Consensus on Physical Health in Patients with Schizophrenia. Eur. Psychiatry.

[3] Miller M.L., Strassnig M.T., Bromet E., Depp C.A., Jonas K., Lin W., Moore R.C., Patterson T.L., Penn D.L., Pinkham A.E. (2021). Performance-Based Assessment of Social Skills in a Large Sample of Participants with Schizophrenia, Bipolar Disorder and Healthy Controls: Correlates of Social Competence and Social Appropriateness. Schizophr. Res..

[4] Hazlett E.A., Romero M.J., Haznedar M.M., New A.S., Goldstein K.E., Newmark R.E., Siever L.J., Buchsbaum M.S. (2007). Deficient Attentional Modulation of Startle Eyeblink Is Associated with Symptom Severity in the Schizophrenia Spectrum. Schizophr. Res..

[5] Jameel L., Rus-Calafell M., Cella M., Bradley J., Valmaggia L. (2024). A Case Series in Using Virtual-Reality Assisted CBTp for Social Difficulties in Psychosis. J. Behav. Cogn. Ther..

[6] Polese D., Fornaro M., Palermo M., De Luca V., De Bartolomeis A. (2019). Treatment-Resistant to Antipsychotics: A Resistance to Everything? Psychotherapy in Treatment-Resistant Schizophrenia and Nonaffective Psychosis: A 25-Year Systematic Review and Exploratory Meta-Analysis. Front. Psychiatry.

[7] Correll C.U., Schooler N.R. (2020). Negative Symptoms in Schizophrenia: A Review and Clinical Guide for Recognition, Assessment, and Treatment. Neuropsychiatr. Dis. Treat..

[8] Chan K.C.-S., Hui C.L.-M., Suen Y.-N., Lee E.H.-M., Chang W.-C., Chan S.K.-W., Chen E.Y.-H. (2023). Application of Immersive Virtual Reality for Assessment and Intervention in Psychosis: A Systematic Review. Brain Sci..

[9] Miranda B., Rego P.A., Romero L., Moreira P.M. (2024). Application of Immersive VR Serious Games in the Treatment of Schizophrenia Negative Symptoms. Computers.

[10] Grossman M.J., Doell F.K., Watson-Gaze J., Baer L.H., Martins F., Kidd S.A. (2022). Increasing Access to CBT for Psychosis: Development, Feasibility, and Acceptability of a Specialized Outpatient Service. Community Ment. Health J..

[11] Hazell C.M., Hayward M., Cavanagh K., Strauss C. (2016). A Systematic Review and Meta-Analysis of Low Intensity CBT for Psychosis. Clin. Psychol. Rev..

[12] Li M., Patel J., Katapally T.R. (2025). The Impact of Extended Reality Cognitive Behavioral Therapy on Mental Disorders among Children and Youth: A Systematic Review and Meta-Analysis Protocol. PLoS ONE.

[13] Thomas N. (2015). What’s Really Wrong with Cognitive Behavioral Therapy for Psychosis?. Front. Psychol..

[14] Rees S.N., Crowe M., Harris S. (2020). The Lesbian, Gay, Bisexual and Transgender Communities’ Mental Health Care Needs and Experiences of Mental Health Services: An Integrative Review of Qualitative Studies. J. Psychiatr. Ment. Health Nurs..

[15] Kohn L., Christiaens W., Detraux J., De Lepeleire J., De Hert M., Gillain B., Delaunoit B., Savoye I., Mistiaen P., Jespers V. (2022). Barriers to Somatic Health Care for Persons with Severe Mental Illness in Belgium: A Qualitative Study of Patients’ and Healthcare Professionals’ Perspectives. Front. Psychiatry.

[16] Higgins E.T. (1987). Self-Discrepancy: A Theory Relating Self and Affect. Psychol. Rev..

[17] Torous J., Linardon J., Goldberg S.B., Sun S., Bell I., Nicholas J., Hassan L., Hua Y., Milton A., Firth J. (2025). The Evolving Field of Digital Mental Health: Current Evidence and Implementation Issues for Smartphone Apps, Generative Artificial Intelligence, and Virtual Reality. World Psychiatry.

[18] Yee J., Matheson H., Bogie B.J.M., Du Perron É., Thérond A., Charest M., Van Driel C., Goyette M., Lei Y.T., Noël C. (2025). Cognitive Remediation for Psychosis in Virtual Reality (ThinkTactic VR): A Qualitative, Iterative, User-Centered Co-Development Study. JMIR Ment. Health.

[19] Rizzo A.S., Koenig S.T., Talbot T.B. (2018). Clinical Virtual Reality: Emerging Opportunities for Psychiatry. FOCUS J. Lifelong Learn. Psychiatry.

[20] Nwokedi N.C.N., Soyege N.O.S., Balogu N.O.D., Mustapha N.A.Y., Tomoh N.B.O., Mbata N.A.O., Iguma N.D.R. (2024). Virtual Reality (VR) and Augmented Reality (AR) in Medicine: A Review of Clinical Applications. Int. J. Sci. Res. Sci. Eng. Technol..

[21] Kritikos J., Alevizopoulos G., Koutsouris D. (2021). Personalized Virtual Reality Human-Computer Interaction for Psychiatric and Neurological Illnesses: A Dynamically Adaptive Virtual Reality Environment That Changes According to Real-Time Feedback from Electrophysiological Signal Responses. Front. Hum. Neurosci..

[22] Sarantopoulos A., Alevizopoulos A., Vasiliades J., Alevizopoulos G., Stergiou A., Kritikos I. (2025). Enhancing Cognitive Screening for Alzheimer’s Disease: Integrating Virtual Reality and AI-Driven Speech. Adv. Artif. Intell. Robot. Res..

[23] Lee E.E., Torous J., De Choudhury M., Depp C.A., Graham S.A., Kim H.-C., Paulus M.P., Krystal J.H., Jeste D.V. (2021). Artificial Intelligence for Mental Health Care: Clinical Applications, Barriers, Facilitators, and Artificial Wisdom. Biol. Psychiatry Cogn. Neurosci. Neuroimaging.

[24] Becerra X., Palm A., Haffajee R.L., Contreras J., Barkoff A., O’Connell D., Valdez R.O., Walensky R.P., Brooks-LaSure C., Califf R.M. (2022). Addressing the Nation’s Behavioral Health Crisis: An HHS Roadmap to Integrate Behavioral Health. Forefr. Group.

[25] Ballout S. (2025). Trauma, Mental Health Workforce Shortages, and Health Equity: A Crisis in Public Health. Int. J. Environ. Res. Public Health.

[26] Kang M., Chai K. (2022). Wearable Sensing Systems for Monitoring Mental Health. Sensors.

[27] Koorathota S., Ma J.L., Faller J., Hong L., Lapborisuth P., Sajda P. (2023). Pupil-Linked Arousal Correlates with Neural Activity Prior to Sensorimotor Decisions. J. Neural Eng..

[28] Kritikos J., Tzannetos G., Zoitaki C., Poulopoulou S., Koutsouris D. (2019). Anxiety Detection from Electrodermal Activity Sensor with Movement; Interaction during Virtual Reality Simulation. 2019 9th International IEEE/EMBS Conference on Neural Engineering (NER).

[29] Caravas P., Kritikos J., Alevizopoulos G., Koutsouris D. (2021). Participant Modeling: The Use of a Guided Master in the Modern World of Virtual Reality Exposure Therapy Targeting Fear of Heights. International Conference on Wearables in Healthcare.

[30] Alevizopoulos A., Kritikos J., Alevizopoulos G. (2021). Intelligent Machines and Mental Health in the Era of COVID-19. Psychiatriki.

[31] Page M.J., McKenzie J.E., Bossuyt P.M., Boutron I., Hoffmann T.C., Mulrow C.D., Shamseer L., Tetzlaff J.M., Akl E.A., Brennan S.E. (2021). The PRISMA 2020 Statement: An Updated Guideline for Reporting Systematic Reviews. BMJ.

[32] Tandon R., Gaebel W., Barch D.M., Bustillo J., Gur R.E., Heckers S., Malaspina D., Owen M.J., Schultz S., Tsuang M. (2013). Definition and Description of Schizophrenia in the DSM-5. Schizophr. Res..

[33] Martingano A.J., Hererra F., Konrath S. (2021). Virtual Reality Improves Emotional but Not Cognitive Empathy: A Meta-Analysis. Technol. Mind Behav..

[34] Craig T.K., Rus-Calafell M., Ward T., Leff J.P., Huckvale M., Howarth E., Emsley R., Garety P.A. (2017). AVATAR Therapy for Auditory Verbal Hallucinations in People with Psychosis: A Single-Blind, Randomised Controlled Trial. Lancet Psychiatry.

[35] Garety P.A., Edwards C.J., Jafari H., Emsley R., Huckvale M., Rus-Calafell M., Fornells-Ambrojo M., Gumley A., Haddock G., Bucci S. (2024). Digital AVATAR Therapy for Distressing Voices in Psychosis: The Phase 2/3 AVATAR2 Trial. Nat. Med..

[36] Dellazizzo L., Potvin S., Phraxayavong K., Dumais A. (2021). One-Year Randomized Trial Comparing Virtual Reality-Assisted Therapy to Cognitive–Behavioral Therapy for Patients with Treatment-Resistant Schizophrenia. Schizophrenia.

[37] Du Sert O.P., Potvin S., Lipp O., Dellazizzo L., Laurelli M., Breton R., Lalonde P., Phraxayavong K., O’Connor K., Pelletier J.-F. (2018). Virtual Reality Therapy for Refractory Auditory Verbal Hallucinations in Schizophrenia: A Pilot Clinical Trial. Schizophr. Res..

[38] Smith L.C., Vernal D.L., Mariegaard L.S., Christensen A.G., Jansen J.E., Schytte G., Stokbro L.A., Albert N., Christensen M.J., Thomas N. (2025). Immersive Virtual Reality-Assisted Therapy Targeting Persistent Auditory Verbal Hallucinations in Patients Diagnosed with Schizophrenia Spectrum Disorders in Denmark: The Challenge Assessor-Masked, Randomised Clinical Trial. Lancet Psychiatry.

[39] Jeon E., Li L., Le T.-H., Kim W.-S., Odkhuu S., Kang C.Y., Setiani A., Rami F.Z., Chung Y.-C. (2025). Virtual Reality Therapy Targeting Ideas of Reference in Patients with Psychosis: A Single-Blind Parallel-Group Randomized Controlled Trial. Psychol. Med..

[40] Freeman D., Bradley J., Antley A., Bourke E., DeWeever N., Evans N., Černis E., Sheaves B., Waite F., Dunn G. (2016). Virtual Reality in the Treatment of Persecutory Delusions: Randomised Controlled Experimental Study Testing How to Reduce Delusional Conviction. Br. J. Psychiatry.

[41] Freeman D., Lister R., Waite F., Galal U., Yu L.-M., Lambe S., Beckley A., Bold E., Jenner L., Diamond R. (2023). Automated Virtual Reality Cognitive Therapy versus Virtual Reality Mental Relaxation Therapy for the Treatment of Persistent Persecutory Delusions in Patients with Psychosis (THRIVE): A Parallel-Group, Single-Blind, Randomised Controlled Trial in England with Mediation Analyses. Lancet Psychiatry.

[42] Jeppesen U.N., Vernal D.L., Due A.S., Mariegaard L.S., Pinkham A.E., Austin S.F., Vos M., Christensen M.J., Hansen N.K., Smith L.C. (2025). Virtual Reality-Based versus Standard Cognitive Behavioral Therapy for Paranoia in Schizophrenia Spectrum Disorders: A Randomized Controlled Trial. Nat. Med..

[43] Monaghesh E., Farhang S., Samad-Soltani T. (2025). Virtual Reality Assisted Cognitive Behavioral Therapy Improves Theory of Mind and Decreases Paranoia in Patients with Schizophrenia: A Randomized Controlled Trial. J. Behav. Ther. Exp. Psychiatry.

[44] Pot-Kolder R.M.C.A., Geraets C.N.W., Veling W., Van Beilen M., Staring A.B.P., Gijsman H.J., Delespaul P.A.E.G., Van Der Gaag M. (2018). Virtual-Reality-Based Cognitive Behavioural Therapy versus Waiting List Control for Paranoid Ideation and Social Avoidance in Patients with Psychotic Disorders: A Single-Blind Randomised Controlled Trial. Lancet Psychiatry.

[45] Van Der Stouwe E.C.D., Geraets C.N.W., Berkhof M., Hidding M., Van Amstel S., Van Den Berg D., Van Grunsven R., De Jager J., Kooijmans E., Sageot M. (2025). Virtual-Reality Cognitive Behavior Therapy versus Cognitive Behavior Therapy for Paranoid Ideation: A Pragmatic, Single-Blind, Multicenter Randomized Clinical Superiority Trial. Psychol. Med..

[46] Sterne J.A.C., Savović J., Page M.J., Elbers R.G., Blencowe N.S., Boutron I., Cates C.J., Cheng H.-Y., Corbett M.S., Eldridge S.M. (2019). RoB 2: A Revised Tool for Assessing Risk of Bias in Randomised Trials. BMJ.

[47] McGuinness L.A., Higgins J.P.T. (2020). Risk-of-bias Visualization (Robvis): An R Package and Shiny Web App for Visualizing Risk-of-bias Assessments. Res. Synth. Methods.

[48] Smith L.C., Mateos A.C., Due A.S., Bergström J., Nordentoft M., Clemmensen L., Glenthøj L.B. (2024). Immersive Virtual Reality in the Treatment of Auditory Hallucinations: A PRISMA Scoping Review. Psychiatry Res..

[49] Aali G., Kariotis T., Shokraneh F. (2020). Avatar Therapy for People with Schizophrenia or Related Disorders. Cochrane Libr..

[50] Fernández-Caballero A., Navarro E., Fernández-Sotos P., González P., Ricarte J.J., Latorre J.M., Rodriguez-Jimenez R. (2017). Human-Avatar Symbiosis for the Treatment of Auditory Verbal Hallucinations in Schizophrenia through Virtual/Augmented Reality and Brain-Computer Interfaces. Front. Neuroinformatics.

[51] Leff J., Williams G., Huckvale M., Arbuthnot M., Leff A.P. (2013). Avatar Therapy for Persecutory Auditory Hallucinations: What Is It and How Does It Work?. Psychosis.

[52] McCarthy-Jones S., Trauer T., Mackinnon A., Sims E., Thomas N., Copolov D.L. (2012). A New Phenomenological Survey of Auditory Hallucinations: Evidence for Subtypes and Implications for Theory and Practice. Schizophr. Bull..

[53] Haddock G., McCARRON J., Tarrier N., Faragher E.B. (1999). Scales to Measure Dimensions of Hallucinations and Delusions: The Psychotic Symptom Rating Scales (PSYRATS). Psychol. Med..

[54] Woodward T.S., Jung K., Hwang H., Yin J., Taylor L., Menon M., Peters E., Kuipers E., Waters F., Lecomte T. (2014). Symptom Dimensions of the Psychotic Symptom Rating Scales in Psychosis: A Multisite Study. Schizophr. Bull..

[55] Steel C., Garety P.A., Freeman D., Craig E., Kuipers E., Bebbington P., Fowler D., Dunn G. (2007). The Multidimensional Measurement of the Positive Symptoms of Psychosis. Int. J. Methods Psychiatr. Res..

[56] Christensen M.J., Rydborg M.P., Jørgensen R., Nielsen C.D., Mainz J., Bell I.H., Thomas N., Smith L.C., Mariegaard L.S., Ward T. (2025). Immersive Virtual Reality–Assisted Therapy for Distressing Voices in Psychosis: Qualitative Study of Participants’ and Therapists’ Experiences in the Challenge Trial. JMIR Serious Games.

[57] Fekete J.T., Győrffy B. (2025). MetaAnalysisOnline.Com: Web-Based Tool for the Rapid Meta-Analysis of Clinical and Epidemiological Studies. J. Med. Internet Res..

[58] Green C.E.L., Freeman D., Kuipers E., Bebbington P., Fowler D., Dunn G., Garety P.A. (2007). Measuring Ideas of Persecution and Social Reference: The Green et al. Paranoid Thought Scales (GPTS). Psychol. Med..

[59] Freeman D., Loe B.S., Kingdon D., Startup H., Molodynski A., Rosebrock L., Brown P., Sheaves B., Waite F., Bird J.C. (2019). The Revised Green et al., Paranoid Thoughts Scale (R-GPTS): Psychometric Properties, Severity Ranges, and Clinical Cut-Offs. Psychol. Med..

[60] Brown P., Spronck P., Powell W. (2022). The Simulator Sickness Questionnaire, and the Erroneous Zero Baseline Assumption. Front. Virtual Real..

[61] Spark J., Pot-Kolder R., Dzafic I., Nelson B., Byrne L.K., Lum J.A.G. (2025). Virtual Reality for the Treatment of Positive Symptoms of Psychosis: A Meta-Analysis of Trials. Curr. Treat. Options Psychiatry.

[62] Cobandag M., Sigala N. (2025). The Effectiveness of Non-Pharmacological Treatments for Auditory Verbal Hallucinations in Schizophrenia Spectrum Disorders: A Systematic Review and Meta-Analysis. Eur. Psychiatry.

[63] Hsu T.-W., Tseng P.-T., Hsu C.-W., Yang F.-C., Changchien T.-C., Lin Y.-H., Liang C.-S. (2025). AVATAR Therapy for Medication-Resistant Auditory Hallucination in Patients with Psychosis: A Systematic Review and Meta-Analysis. Schizophrenia.

[64] Lindgren M., Torniainen-Holm M., Heiskanen I., Voutilainen G., Pulkkinen U., Mehtälä T., Jokela M., Kieseppä T., Suvisaari J., Therman S. (2018). Theory of Mind in a First-Episode Psychosis Population Using the Hinting Task. Psychiatry Res..

[65] Mevlevioğlu D., Tabirca S., Murphy D. (2023). Anxiety Classification in Virtual Reality Using Biosensors: A Mini Scoping Review. PLoS ONE.

[66] Williams L.M., Pines A., Goldstein-Piekarski A.N., Rosas L.G., Kullar M., Sacchet M.D., Gevaert O., Bailenson J., Lavori P.W., Dagum P. (2017). The ENGAGE Study: Integrating Neuroimaging, Virtual Reality and Smartphone Sensing to Understand Self-Regulation for Managing Depression and Obesity in a Precision Medicine Model. Behav. Res. Ther..

[67] Zhao X., Lou Z., Shah P.T., Wu C., Liu R., Xie W., Zhang S. (2025). Integration of Multi-Modal Biosensing Approaches for Depression: Current Status, Challenges, and Future Perspectives. Sensors.

